# Supplementary material for: Inhibition of excessive mitophagy by N-acetyl-L-tryptophan confers hepatoprotection against Ischemia-Reperfusion injury in rats
Source: PeerJ. 2020 Apr 9;8:e8665. doi: 10.7717/peerj.8665 (PMC7151751; doi:10.7717/peerj.8665)
Supplement: Supplemental Information 1 [file peerj-08-8665-s001.docx]

| Gene | Sequence |
| --- | --- |
| *Beclin1* | F:5’-AGGAGTTGCCGTTGTACTGTTCTG-3’ |
|  | R:5’-TGCCTCCAGTGTCTTCAATCTTGC-3’ |
| *LC3-Ⅱ* | F:5’-TCGCCGACCGCTGTAAGGAG-3’ |
|  | R:5’-CGCCGGATGATCTTGACCAACTC-3’ |
| *ATG-7* | F:5’-GTGAACCTCAGCGGATGTATGGAC-3’ |
|  | R:5’-CCAGCAGCAGGCACTTGACAG-3’ |
| *P62* | F:5’-GTGAACCTCAGCGGATGTATGGAC-3’ |
|  | R:5’-CCAGCAGCAGGCACTTGACAG-3’ |
| *mtAtp6* | F:5’-GCCGTAATTCTAGGCTTCCGACAC-3’ |
|  | R:5’-TGCTGTTAGTCGTACTGCTAGTGC-3’ |
| *Rp113* | F:5’-ACCTGCACAGAAGAACGATGATGC-3’ |
|  | R:5’-TGTACTCGTCCAGCCGCTTAGG-3’ |
| *ND1* | F:5’-GTCCTCCTAATAAGCGGCTCCTTC-3’ |
|  | R:5’-GGTCCTGCGGCGTATTCGAC-3’ |
| *COX-1* | F:5’-ATCTCACTTACGGCCGTCCT-3’ |
|  | R:5’-GGGGCATCCATGCAGTCATT-3’ |
| *GAPDH* | F:5’-TGATTCTACCCACGGCAAGTT-3’ |
|  | R:5’-TGATGGGTTTCCCATTGATGA-3’ |
