# Supplementary figures and images for: Inhibition of excessive mitophagy by N-acetyl-L-tryptophan confers hepatoprotection against Ischemia-Reperfusion injury in rats
Source: PeerJ. 2020 Apr 9;8:e8665. doi: 10.7717/peerj.8665 (PMC7151751; doi:10.7717/peerj.8665)

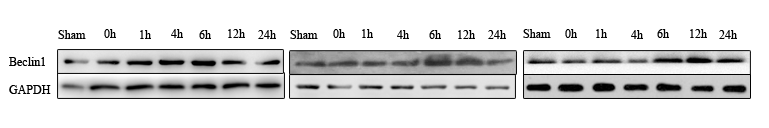

Supplement: Supplemental Information 4 [file peerj-08-8665-s004.zip › Western Blot/Figure 1/图片1.png]

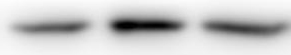

Supplement: Supplemental Information 4 [file peerj-08-8665-s004.zip › Western Blot/Figure 2/ATG7 WB.jpg]

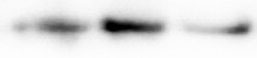

Supplement: Supplemental Information 4 [file peerj-08-8665-s004.zip › Western Blot/Figure 2/Beclin1.jpg]

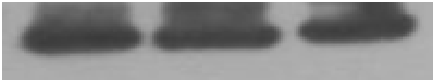

Supplement: Supplemental Information 4 [file peerj-08-8665-s004.zip › Western Blot/Figure 2/GAPDH.png]

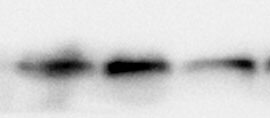

Supplement: Supplemental Information 4 [file peerj-08-8665-s004.zip › Western Blot/Figure 2/LC3.jpg]

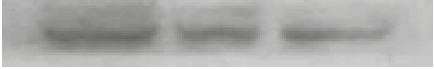

Supplement: Supplemental Information 4 [file peerj-08-8665-s004.zip › Western Blot/Figure 2/P62.png]

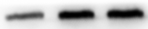

Supplement: Supplemental Information 4 [file peerj-08-8665-s004.zip › Western Blot/Figure 3/ATG7(2).tif]

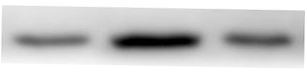

Supplement: Supplemental Information 4 [file peerj-08-8665-s004.zip › Western Blot/Figure 3/Beclin(cell).jpg]

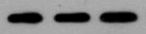

Supplement: Supplemental Information 4 [file peerj-08-8665-s004.zip › Western Blot/Figure 3/GAPDH.jpg]

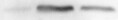

Supplement: Supplemental Information 4 [file peerj-08-8665-s004.zip › Western Blot/Figure 3/LC3.jpg]

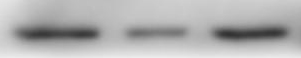

Supplement: Supplemental Information 4 [file peerj-08-8665-s004.zip › Western Blot/Figure 3/P62.jpg]

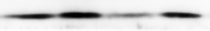

Supplement: Supplemental Information 4 [file peerj-08-8665-s004.zip › Western Blot/Figure 8/3-MA(WB) (1).tif]

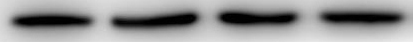

Supplement: Supplemental Information 4 [file peerj-08-8665-s004.zip › Western Blot/Figure 8/GAPDH99.jpg]
